# Glioma extracellular vesicles for precision medicine: prognostic and theragnostic application

**DOI:** 10.1007/s12672-022-00514-0

**Published:** 2022-06-18

**Authors:** Hany E. Marei, Asmaa Althani, Nahla Afifi, Anwarul Hasan, Thomas Caceci, Ingrid Cifola, Sara Caratelli, Giuseppe Sconocchia, Igea D’Agnano, Carlo Cenciarelli

**Affiliations:** 1 Department of Cytology and Histology, Faculty of Veterinary Medicine, Mansoura University, Mansoura, 35116 Egypt; 2 Biomedical Research Center, Qatar University, Doha, Qatar; 3 Qatar Biobank, Doha, Qatar; 4 Department of Mechanical and Industrial Engineering, College of Engineering, Qatar University, Doha, Qatar; 5 Biomedical Sciences, Virginia Maryland College of Veterinary Medicine, Blacksburg, VA USA; 6 Institute for Biomedical Technologies (ITB)-CNR, Segrate, Italy; 7 Institute of Translational Pharmacology (IFT)-CNR, Rome, Italy

**Keywords:** GBM, GSCs, Extracellular vesicles, Prognosis, Diagnosis, Therapy

## Abstract

EV produced by tumour cells carry a diverse population of proteins, lipids, DNA, and RNA molecules throughout the body and appear to play an important role in the overall development of the disease state, according to growing data. Gliomas account for a sizable fraction of all primary brain tumours and the vast majority of brain malignancies. Glioblastoma multiforme (GBM) is a kind of grade IV glioma that has a very dismal prognosis despite advancements in diagnostic methods and therapeutic options. The authors discuss advances in understanding the function of extracellular vesicles (EVs), in overall glioma growth, as well as how recent research is uncovering the utility of EVs in glioma diagnostics, prognostic and therapeutics approaches.

## Introduction

Malignant gliomas, particularly GBM, are the most fatal primary brain tumours due to their highly infiltrative development patterns, with an annual incidence of roughly 3/100,000 [1]. According to the 2016 WHO update, gliomas are classified as contained gliomas (WHO grade I) or diffusely infiltrating gliomas (WHO grade II–IV) based on their development pattern and the presence or absence of an isocitrate dehydrogenase (IDH) mutation [2]. Almost all GBM tumours return after surgery, radiotherapy, and chemotherapy, leaving patients with fewer treatment options and a poor prognosis. Difficulties in tracking therapy responses and tumour progression aggravate this aggressive cancer phenotype. As a result, biomarkers to diagnose and monitor disease burden and therapy responses in GBM patients in a safe, reliable, and timely way, particularly before clinically evident alterations, are urgently needed [3]. New histological, cellular, and genetic methods for characterising GBM are crucial because they will aid in the development of future treatments that are more specific to tumour cells.

**Fig. 1:**
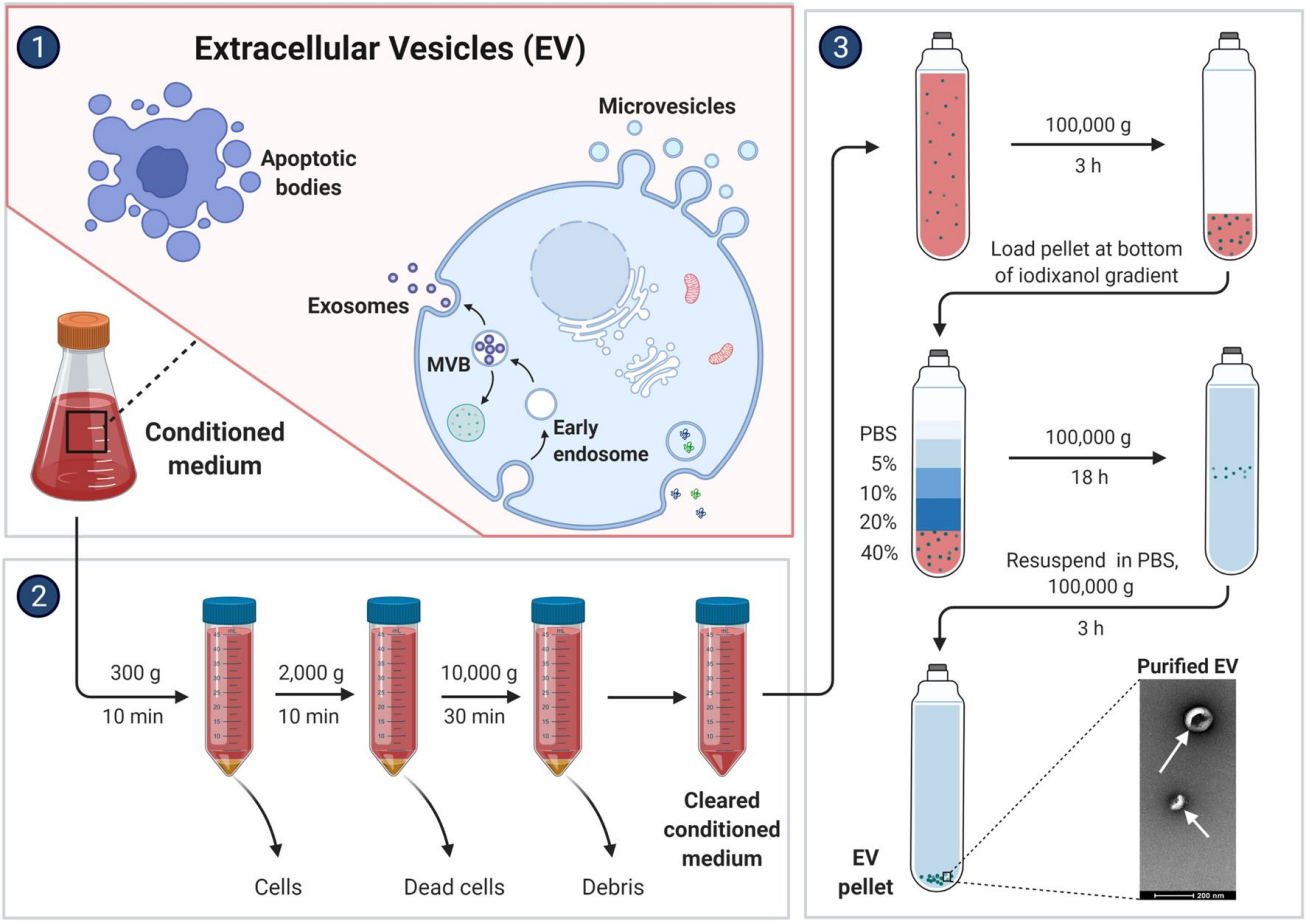
Example of protocol to isolate and purify EVs from conditioned cell culture media. Large EVs (macrovesicles, apoptotic bodies, and oncosomes) derive directly from the extrusion of plasma membranes. Small EVs (also called exosomes) are produced in the multivesicular bodies (MVBs) that are then secreted in the extracellular space, fusing with the plasma membrane. Density gradient ultracentrifugation protocol to isolate EVs involves centrifuging conditioned cell culture media at different speeds ranging from 300 to 10,000 g for 10–30 min, then ultra centrifuging at 100,000*g* for 3 h. The EV pellets are then ultracentrifuged for 18 h at 100,000*g* on an iodixanol gradient to be purified. The pellets containing purified EVs are then resuspended in PSB to be washed ultra centrifuging at 100,000*g* for 3 h

EVs are natural nanoparticles produced from the membrane that are secreted by all cells via various pathways. They come from two separate subcellular membranes, the plasma membrane and endocytic membranes [4, 5]. They are highly heterogeneous and can be divided into small EVs (also known as exosomes, with sizes of 100 or 200 nm) and large EVs (also known as microvesicles (MVs) or ectosomes, with sizes > 200 nm) according to the MISEV (Minimal Information for Studies of Extracellular Vesicles) 2018 guidelines [6]. Intraluminal vesicles are the antecedents of exosomes, which are formed inside cells by the internal budding of the membrane of endocytic cisternae during early endosome development (ILVs). ILVs clump together to form multivesicular endosomes or bodies (MVEs/MVBs), which are secreted and fuse with the cell membrane [7–9]. MVs, on the other hand, come from the plasma membrane’s extroversion, generating blebbing and release into the extracellular area [10] (Fig. 1). Exosomes that are larger than 200 nm and MVs that are smaller than 200 nm exist. There are currently no unique markers that distinguish exosomes from other nanoscale vesicles, which could limit their specific identification in human fluids. Proteins (both soluble and as part of the EV membrane), nucleic acids, lipids, and metabolites make up EV cargo, which can be transported by EVs to local or distant cells. Tumor cells produce large amounts of EVs, whose cargo composition is altered as compared to their healthy counterpart. EVs have been related to the development of cancer because they deliver factors that control tumor progression processes such as cell proliferation, migration, invasion, angiogenesis, and chemoresistance and that are transferred in the cell-to-cell crosstalk within the tumour microenvironment components and between cell populations in distant organs [11–13]. In the past few years EVs have been considered as emerging component of the so-called liquid biopsy, a minimally invasive approach performed on bio-fluids to detect and quantify circulating cells or other cellular components including EVs [14]. The profiling of the EV-cargo in the circulation of GBM patients has given impetus to their putative role as non-invasive cancer biomarkers [15]. In particular, the RNA profiles of EVs reveals that micro RNA (miRNAs) are highly enriched in these EVs and show different signatures between healthy subjects and cancer patients. This has the potential to introduce a groundbreaking approach in early diagnosis of cancer since circulating EV-miRNAs are stable and easily accessible biological molecules and their detection tend to reflect the pathophysiological state of the primary affected tissue. In addition, the use of blood/fluid specimens in the diagnostic routine presents some advantages: samples are easy to obtain without invasive procedures for the patients; molecules (e.g. miRNAs) that are incorporated into EVs to circulate in the blood maintain a highly stable form; samples can be stored thus preserving quality and reproducibility of the analysis; the costs are reduced and there is rising interest in using them as non-invasive indicators for disease diagnosis and disease recurrence monitoring.

Moreover, due to their lipid bilayer membrane, EVs can tolerate degrading environments, and, for this reason, they may be exploited to enhance the concentrations of drugs delivered to the target tumour site. Since some of the cargo constituents might underlie their cellular tropism, EVs could in theory be manipulated into highly selective drug vehicles, hence limiting side effects and toxicity on normal stroma cells in the brain microenvironment.

EV separation is achieved by different protocols [16]. Among them, density gradient ultracentrifugation, involves gradually centrifuging EV-containing liquid (conditioning cell culture medium or biofluids) at different speeds ranging from 300 to 10,000 g for 10–30 min, then 100,000 g for 3 h. To be purified, the EV pellets are then placed onto an iodixanol gradient and centrifuged for 18 h at 100,000*g*. Finally, purified EV pellets are resuspended in PSB/washing and centrifuged at 100,000*g* for 3 h. After that, EVs are cleansed and separated (Fig. 1).

Thus, since EVs produced by GBM cells circulate in patients’ peripheral blood potentially carrying diagnostic molecules [17, 18], further research into the function of EVs in the formation of gliomas, as well as their potential use in diagnostic, prognostic, and therapeutic purposes, is required. As a result, the current study offers an overview of EVs biological composition, their possible involvement in glioma development, and their diagnostic, prognostic, and therapeutic value.

## Classifications and complex mutation burdens of GBM

To ensure individualized brain tumour management, precise classification of the evolutionary pattern of GBM based on its cellular origin rather than histopathological criteria is required [19]. The elucidation of the mutational burdens of GBM has been highlighted and listed in The Cancer Genome Atlas (TCGA) project, where several mutations in oncogenes and tumour suppressor genes have been recorded. This will aid in resolving GBM's high heterogeneity and highlighting the major pathways involved in its pathogenesis and progression [20].

The tumor protein p53 **(**TP53), phosphoinositide 3-kinases (PI3K), and retinoblastoma tumor suppressor protein 1 (Rb1) signaling pathways have all been shown to play important roles in GBM pathogenesis. Mutations in the aforementioned pathways increase the lifespan of GBM cells primarily by inhibiting apoptosis and increasing the cellular proliferation rate [21]. In 2016, the WHO established further improvements in the classifications of brain tumours, including GBM, in which phenotypic and genotypic diagnostic characteristics were integrated with the goal of improving the clinical and therapeutic outcomes of GBM [22]. The WHO classifies GBM into two types based on genetic and mutational makeup: primary and secondary. Primary GBM affects older patients and accounts for 90% of all GBMs. It is distinguished by overexpression of cellular receptors such as epidermal growth factor receptor (EGFR) and platelet derived growth factor receptor (PDGFR), deletion of Cyclin-dependent kinase inhibitor 2A/B (CDKN2A/B), mutations in phosphatase and tensin homolog (PTEN) genes, and mutations in TERT promoters. Secondary GBM is associated with other genetic changes such as isocitrate dehydrogenase 1 **(**IDH1) and TP53 mutations, methylation of the O^6^-methylguanine-DNA methyltransferase (MGMT) promoter, and chromosome 19q loss. It has a better prognosis and is usually diagnosed at a younger age [20].

## Role of EVs in GBM progression

### EVs cargo can promote GBM progression

EVs have been studied extensively in the context of cancer progression in general and GBM in particular. GBM cell-EV cargo, has been demonstrated to carry a number of cancer-modulating effector oncoproteins, oncotranscripts, and microRNAs that are important in the formation and progression of GBM [23–25]. EVs secreted by GBM cells have been related to the transformation of normal brain cells into malignant GBM cells, as well as the production of a tumor-friendly microenvironment by modifying the activity of surrounding stromal cells [26]. The cargo of GBM EVs is crucial in GBM proliferation and invasiveness, in that, for example, EVs, decorated with the L1 adhesion molecule ectodomain (L1CAM), released in the tumor microenvironment stimulate motility and invasion of GBM cells [27]. In addition to GBM tumour cells, the GBM microenvironment contains a subset of stem cells known as glioblastoma stem cells (GSCs). GSCs are unique in that, they can perform two functions, on the one hand, they release EVs, which contain cargo that have protumorigenic effects on GBM cells. On the other hand, are target for EVs released by GBM cells within the microenvironment [28]. The constant flow of EVs to and from GSCs could contribute to GBM heterogeneity, which is a key hallmark of this tumour type [29]. The GBM EVs are also involved in trafficking several other molecules and receptors that have been shown to play crucial role in GBM proliferation and tumor aggressiveness. EVs are thought to be involved in PTEN-modulated trafficking, according to Putz et al. [30]. PTEN is a protein found in the nucleus and cytoplasm of cells that has been linked to cancer aggressiveness. Maintaining a tumor-free state necessitates PTEN-regulated intercellular trafficking via EVs. The Ndfip1 protein aids in the internalisation of PTEN-enriched EVs. Nedd4 family interacting protein 1 (Ndfip1) is inhibited in GBM, which prevents PTEN from accumulating in the nucleus and hence enhances tumour cell survival and proliferation [31]. In addition, EGFRvIII, PDGFR, and HER2 (human epidermal growth factor receptor 2) are important underlying components in GBM proliferation. EVs containing these receptors deliver them to cells that lack this protein complex, promoting signaling pathways involved in cell proliferation, resulting in cancerogenic activity in previously unaffected cells [31].

### Potential role of GBM EVs in induction of chemoresistance

Several studies have devoted to elucidating the potential mechanisms by which GBM cells become resistant to currently available pharmacological drugs. In this regard, it has been reported that GBM contains a small population of cancer stem cells known as GSCs, which have been shown to be resistant to pharmacological substances [32, 33]. EVs derived from GSCs have been shown to induce chemoresistance in recipient normal cells, primarily through the transfer of adenosine-producing enzymes, which increases the adenosine level in their cytoplasm. Elevated adenosine levels are required for the induction of chemoresistance, which is accomplished primarily through the action of the multidrug resistance protein 1 (MRP1) transporter [31]. Specific surface markers for GSCs, such as cluster differentiation 133 (CD133) and cluster differentiation 44 (CD44), have been found inside EVs derived from them, and thus represent promising biological chemoresistance markers [31].

The EVs are also implicated in reduction the level of pharmacological molecules inside the cells mainly through the release of drugs into the extracellular medium and modulating the expression of enzymes involved in drug metabolism and action. EVs formed as a result of temozolomide resistance GBM cells had a high level of MGMT and alkylpurine-DNA-N-glycosylase (APNG), both of which counteract the DNA-damaging effects of alkylating substances [34].

### GBM EVs and their role in enhancing the invasive ability of GBM cells

Another important area that has been linked to the roles of EVs in the progression and development of GBM is invasion. GBM cells have been shown to invade surrounding tissues by extending membrane-derived extensions known as invadopodia, which degrade the surrounding matrix primarily through the release of proteolytic factors [35, 36]. Recent research has revealed that invadopodia are derived not only from GBM cells but also from their associated astrocytes, and that GBM EVs may modulate the astrocytes’ phenotype toward tumorigenicity or acquisition of GBM-supporting properties [37, 38]. A reciprocal role of activation between invadopodia and GBM EVs has been described, in which invadopodia stimulate EV release from GBM cells and EVs trigger invadopodia synthesis and maturation [39], primarily through the release of several proteins derived from GBM EVs such as Annexin A1 (ANXA1), integrin beta 1 (ITGB1), and actin-related protein 3 (ACTR3) [28].

### GBM EVs and angiogenesis

As previously stated, the presence of GSCs is a key factor that contributes to the invasiveness and chemoresistance properties of GBM [24]. GSCs have been discovered to be strategically placed near vascular niches. This would allow the GSC to be sustained by an adequate supply of oxygenated blood while delivering a slew of proangiogenic factors that stimulate angiogenesis [24, 26]. Furthermore, GBM-derived EVs have been found to be rich in several pro-angiogenic factors including vascular endothelial growth factor (VEGF), transforming growth factor beta 1 (TGF-β1), C-X-C chemokine receptor type 4 (CXCR4), plasminogen activator and proteases [31]. The pro-angiogenic VEGF released by GSCs and transported by GBM-derived EVs increases the aggressive nature of GBM [31]. Other GBM EVs-derived substances such as TGF-β1, are important in glioma extracellular matrix modification and remodeling, as well as increasing the proliferative potential of glioma-associated endothelial cells [40]. GBM cell proliferation, VEGF synthesis, and endothelial tube formation are all stimulated by CXCR4 generated from GBM EVs, and the C-X-C Motif Chemokine Ligand 12 (CXCL12) ligand enables reciprocal communication between GBM and endothelial cells [31, 41]. Induction and activation of angiogenesis require additional factors such as plasminogen activator representatives and matrix-metalloproteinase [40]. MicroRNA 21 (MiR21)-enriched EVs activate the VEGF pathway and increase endothelial cell proliferation [42], whereas miR-1-rich EVs reduce the pro-angiogenic impact and thereby limit GBM-associated angiogenesis [31]. Other factors, such as semaphorin-3A, a pro-permeability factor, enhance vascular permeability in GBM [43].

### Role of EV-associated microRNA in GBM progression

Micro-RNAs (also called miR or miRNA) are single-stranded RNAs with a short sequence that regulate gene expression that were discovered in GBM-derived EVs. They interact with signaling pathways involved in cell viability and the cellular life cycle to increase the viability of tumour cells [44]. Several miRs (miR-21, miR-29a, miR-221, and miR-222) have been implicated in enhancing tumour cell proliferation and blocking apoptosis in GBM cells, according to in vitro studies and microarray analysis [31]. Other molecules found within GBM-derived EVs, such as miR-451, help glioma cells adapt to metabolic stress [45]. Hypoxia-induced EVs also aid GBM invasion and migration by altering the extracellular matrix structure and interacting with neighboring cells via the proteins they carry [46]. (Table 1).

**Table 1:** A table summarizing the most important EV-associated biomarkers and their potential clinical implications

EV-associated biomarkers	Clinical implications	References
ANXA1, ITGB1, ACTR3	Stimulate invadopodia and provide invasive capacity	Matarredona et al. [28]
miR-5096	Suppression Kir4.1 expression	Hoshino et al. 2015 [39]
miR-21, miR-29a, miR-221, and miR-222	Enhance cell proliferation and apoptosis inhibition	Quezada et al. [31]
Ndfip1	Enhances tumour cell survival and proliferation	Quezada et al. [31]
EGFRvIII, PDGFR, and HER2	Promoting signaling pathways involved in cell proliferation	Quezada et al. [31]
PTEN mutations	Promote excessive tumour proliferation	Montemurro et al. [20]
TERT promoter	Induces excessive tumour proliferation	Montemurro et al. [20]
EGFR/EGFRvIII	Diagnostic marker. Poor prognosis	Montemurro et al. [20]
TGF-β1	Increasing the proliferative potential of glioma-associated endothelial cells	Giusti et al. [40]
VEGF, TGF-β1, CXCR4	Stimulate angiogenesis	Quezada et al. [31]
miR-451	Help glioma cells adapt to metabolic stress	Godlewski et al., 2010 [45]
IDH1 and TP53 mutations, methylation of the MGMT promoter	It has a better prognosis and is usually diagnosed at a younger age	Montemurro et al. [20]
miR-451	Help glioma cells adapt to metabolic stress	Godlewski et al. [45]
CD133, CD44	Represent promising biological chemoresistance markers	Quezada et al. [31]
IDH-1 mutant	Diagnostic marker	Louis et al. [2]
PD-L1	Inhibition of T cell proliferation and activation and subsequent downmodulationn of immunological assaults on tumour cells	Litak et al. [53]

*ANXA1* annexin A1; *ITGB1* integrin beta-1; *ACTR3* actin-related protein 3; *miR-5096* microRNA 5096; *miR-21* microRNA 21; *miR-29a* microRNA 29a; *miR-221* microRNA 221; *miR-222* microRNA 222; *miR-451* microRNA 451; *Ndfip1* Nedd4 family interacting protein 1; *EGFRvIII* epidermal growth factor receptor/epidermal growth factor receptor variant III; *PDGFR* platelet drived growth factor receptor; *HER2* human epidermal growth factor 2; *PTEN* phosphatase and tensin homolog; *TERT* telomerase reverse transcriptase; *TGF-β1* transforming growth factor-beta 1; *VEGF* vascular endothelial growth factor; *CXCR4* C–X–C motif chemokine receptor 4; *miR-451* microRNA 452; *CD133* cluster differentiation 133; *CD44* cluster differentiation 44; *IDH-1* isocitrate dehydrogenase 1; *PD-L1* programmed death-ligand 1

Nonetheless, miR-451 has a distinctive function that is greatly influenced by the metabolic state of the environment. Its overexpression causes the calcium binding Protein 39 / Liver kinase B1/AMP-activated protein kinase (CAB39/LKB1/AMPK) pathway to be repressed, which enhances the rate of cancer cell proliferation [44]. Another plausible mechanism under investigation is if decreasing miR-451 promotes AMPK activity, which could provide an alternate explanation for GBM’s strong invasive capacity [44] (Table 1).

According to Van der Vos et al. EVs generated by GBMs cause a remodeling of both the cytoskeleton and the inflammatory characteristics of monocytes, which increases immunological tolerance to the tumour [47], whereas EVs generated by GBMs cause a remodeling of both the cytoskeleton and the inflammatory characteristics of monocytes, according to Gabrusiewicz et al. [48] who found that this enhances immunological function. The reversal of this process, which requires loading anti-sense miR nucleotides into EVs to target GBM cells and convert their phenotype to chemo sensitive [31], could give researchers with a wealth of new data on chemoresistance-inducing miR. Kir4.1 expression is suppressed by MiR-5096, which is present in GBM-derived EVs, resulting in increased filopodia outgrowth. It also promotes the production of more EVs, which increases their transfer to neighboring cells and speeds up GBM invasion. EVs and invadopodia have a mutual and synergistic interaction, according to Hoshino et al. [39]: whereas invadopodia stimulate EV release, EVs play a significant role in invadopodia creation and maturation.

### Role of EV in modulation GBM microenvironment and immune response

GBM, as previously stated, uses a variety of communication methods to hijack the basic activities of non-tumoural cells in order to support tumour invasion, with the generation of EVs being one of the most important. Innate immune system cells such as microglia, monocytes, and macrophages are among the most common cells in the GBM microenvironment, and tumor-associated macrophages (TAMs) are made up of them.

The GBM microenvironment is made up of tumour cells, immune cells (such as monocytes, macrophages, and T cells), GSCs, endothelial cells, neurons, astrocytes, and oligodendrocytes, as well as extracellular matrix components [49].

De Vrij et al. [50] recently demonstrated that EVs derived from GBM can alter the TAM phenotype from pro- to anti-inflammatory, hence promoting tumour growth. M1 macrophages are transformed into M2 macrophages by GBM-released EVs, which are incapable of killing invading tumour cells but can protect tissue integrity [51]. They also prevent monocytes from converting into immunologically active macrophages [31]. EVs also boost macrophage phagocytic activity, which leads to extracellular matrix degradation and tumour cell movement [28].

Patients with GBM have a weaker immunological response, which is reflected in changes in the circulating lymphocyte ratio and immune modulation via an aberrant T helper type 2 lymphocyte (Th2) pathway. While T helper (CD4 +) cells are in lower numbers than in the general population, lymphocyte regulators are in high numbers, resulting in insufficient cell immunity [31]. T helper type 1 lymphocyte (Th1) is in charge of the general anti-tumour immune response. Both cytokines and EVs, on the other hand, generate a Th2 immune response in GBM patients, which promotes M2 macrophages to release anti-inflammatory molecules, hence promoting GBM formation [31].

T-cell function is further suppressed by check point molecules carried by EVs that compromise anti-cancer immunity, such as programmed death-ligand 1 (PD-L1) [52]. The PD-1 protein protects against autoimmunity by keeping the immune system in check. GBM cells induce PD-L1 secretion and activation of PD-1–PD-L1 pathway in microglia, which results in the inhibition of T cell proliferation and activation and subsequent downmodulation of immunological assaults on tumour cells [53]. According to Domenis et al., the suppression of T-cell immune response by GSC-secreted EVs is mediated by monocytic myeloid-derived suppressor cells (Mo-MDSC) [54]. In addition, Huang et al. [55] discovered that tenascin C, an important component of the extracellular matrix, plays an important role in GBM by maintaining GSC stemness and preventing T cell activation and migration [56]. Surprisingly, the size of a tumour has an impact on T cell immunology. According to Brooks et al. [57], T lymphocyte mitotic capability is recovered after GBM surgical excision, but it is reduced if the tumour recurs.

## Potential diagnostic role of GBM EVs

The essential features that characterize a good diagnostic marker are bioavailability, ease of isolation, and the ability to consistently convey vital information about the illness status. It's no surprise, then, that tumor-derived EVs have gotten a lot of attention as a potential diagnostic tool in the last decade, because they essentially act as nano-scaled parcels containing a variety of molecules, some of which have already been shown to have great diagnostic potential, but which lack one or both of the first two criteria mentioned above. Non-invasive or minimally invasive diagnostics have a number of advantages over invasive diagnostic methods, including speed, cost, and patient acceptability. EVs, for example, have been discovered in breast milk, plasma, cerebrospinal fluid (CSF), urine, and saliva, among other physiological fluids [17, 58–60]. Advances in EV isolation [61] have suggested that EVs could be used as a new minimally invasive diagnostic tool. The reason for using EVs for GBM and other tumour diagnostics can be attributed mostly to their distinct protein and nucleic acid payloads, which are specific to the tumour cells from which they are separated and can be employed as biomarkers for disease diagnosis.

Glioma initiation and progression have been linked to miRNAs, which are small noncoding RNA species that influence gene expression at the posttranscriptional level [62]. The miRNA that has been targeted for diagnostic purposes is a major component of the GBM EV cargo. miRNAs appear to have taken the lead in diagnostic research. In this regard, cancer cells' EVs have been identified as major carriers of oncogenic miRNAs [63]. Quantification of miRNA levels in human saliva and serum samples revealed that the majority of these miRNAs are present as exosomal cargo [64]. MiRNA sequestration within EVs has been discovered to be critical for their preservation by nuclease degradation in the blood and other body fluids [65]. The EV-contained miR-21was one of the first miRNAs to be indicated for GBM patients diagnostic purposes [66].

At the level of protein biomarkers for GBM EVs, the EGFRvIII (the most common oncogenic variant of the receptor for GBM) has been demonstrated in EVs, including EVs generated by glioma cells, and cell lines transfected with EGFRvIII [67]. EVs produced by cells that expressed the mutant receptor, as well as EVs detected in GBM patients' serum, all had the mutant receptor on their surfaces [68]. In glioma cell lines, HSPs such as HSP60, HSP70, and HSP90 have been linked to a variety of pathways involved in cell proliferation, survival, invasiveness, and migration. HSPs have been reported to be carried as a continuous cargo by EVs and other EVs. As a result, EVs derived from glioma cells with a high concentration of HSPs have a strong diagnostic potential [69].

## Potential use of EVs for GBM therapy

There are still certain roadblocks in the current GBM treatment that are preventing the creation of a successful therapeutic strategy. Surgery, radiation therapy, and temozolomide (TMZ) chemotherapy are currently the primary treatments for GBM [70]. Unfortunately, the surviving tumour cells begin to overexpress MGMT over time, conferring a high level of resistance to TMZ. While the usual GBM therapy protocol may help to enhance patients’ disease-free survival, overall patient survival is not considerably improved [71]. The use of MGMT inhibitors is one potential way to combat TMZ increasing resistance; however, the high risk of disrupting the DNA repair machinery in healthy cells has prevented their widespread usage [72]. The use of antiangiogenic medications like bevacizumab in combination with TMZ has resulted in a longer period of disease-free time [72]. The presence of multiple resistance mechanisms, the invasive nature of GBM tumor cells, their genetic heterogeneity, and the presence of the blood–brain barrier are the most critical factors found to diminish the efficacy of the currently standard therapy strategy for GBM [73–75].

Nanocarriers or tumour targeting vesicles have recently been developed for effective and targeted drug delivery to tumour sites [76, 77]. Despite the fact that nanocarriers have been shown to be successful for targeted therapy and in crossing the BBB [78], the artificial nature of nanoparticles and their lack of biocompatibility with tumour and healthy tissues pose a significant risk to their use in human cancer therapy [79]. The risk associated with the use of synthetic nanocarriers have prompted oncologists to look for an alternative carrier that can not only deliver targeted therapy and cross the BBB, but also has a high level of biocompatibility. Natural derived EVs appear suitable for this purpose. The capacity of EVs to penetrate deeply into tissues, have a long circulation half-life, evade the immune system, and target specific tumour cell subtypes clearly qualifies them as a valid alternative to synthetic, non-biocompatible nanoparticle drug delivery systems [80].

The unique structure of EVs, which consists of a hydrophobic outer shell and a hydrophilic aqueous interior, makes them ideal for delivering diverse cargoes to tumour cells. EVs containing specified cargo have been shown to be uploaded using both passive (incubation) and active (electroporation) delivery methods. While active approaches tend to be more efficient in terms of loading efficiency, they may be associated with a higher risk of compromising the integrity of EV membranes [81].

EVs can be pay loaded with different compounds including peptides, small molecules, and siRNAs, among other therapeutic agents. Incorporation of drugs like TMZ into EVs has been shown to improve its effectiveness in targeting GBM cells. This could protect against TMZ resistance, which has been shown to be caused by Pglycoprotein (Pgp) overexpression [82]. EV-based drug delivery appears to be able to bypass the Pgp drug efflux pump (precise mechanism uncertain), thus lowering the required dosage of the drug [83].

The capacity of EVs to specifically engage with target cells once delivered into the body is one essential features for effective targeting of specific cell types, including tumour cells. EVs from zebra fish brain endothelial cells payloaded with paclitaxel and doxorubicin have been demonstrated to not only cross the blood brain barrier (BBB), but also to target GBM cells with high specificity [84].

**Fig. 2:**
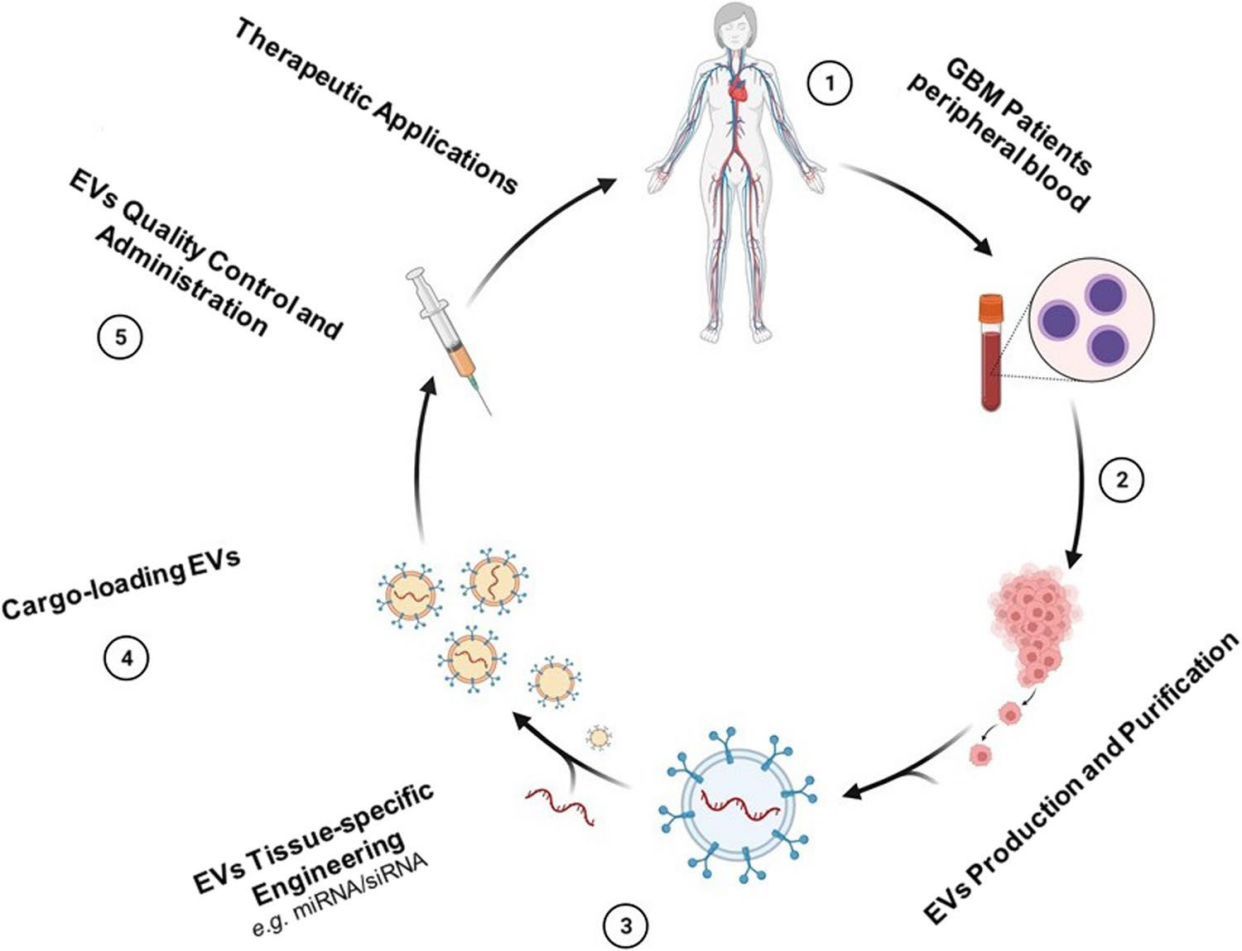
Self-derived GBM EVs for therapeutic applications. EVs are extracted from patient blood. EVs could be engineered to express tissue-specific protein. Diverse cargo, such as miRNA, antagomir, siRNA, and drugs, can be pay loaded in the EVs. Following standardizing protocols and quality controls, EVs could be administrated to GBM patients to target GBM cancer cells

Several ways have been implemented to boost the selectivity of EVs in targeting tumour or diseased cells. EVs derived from patient-specific tumour cells (Fig. 2) were discovered to have a natural inclination to target tumour cell types [84]. Although, in order to establish this paradigm, more research will be required. EV target specificity has been found to be considerably improved when EVs are engineered to express specific targeting proteins on their surface during EV synthesis. EVs with rabies virus glycoprotein (RVG)–LAMP2B fusion proteins on their surfaces were able to traverse the BBB and target acetylcholine receptor-expressing brain cells [85]. The production of genetically modified cells that can produce a specific set of EVs that can be used to target a specific disease such as Parkinson’s disease has been the focus of recent research on the use of EVs for therapeutic reasons [86].

EVs could be developed to act as a potential glioma vaccination, in addition to its usefulness as a medication delivery vehicle. Because EVs originating from individual tumour cells display the same tumour antigen specific for that tumour, providing such tumor-specific EVs can activate dendritic cells (DCs), which can then activate CD8 + T cells’ antitumor potential [87]. When tumour peptide-containing EVs were treated with DCs, they were found to increase CD8 + T cell activity and decrease tumour progression [88].

The presence of major histocompatibility complex-1 **(**MHC-1) molecules on the surface of patient-derived EVs may imply the feasibility of using patients-specific EVs as a prospective treatment method for various tumours, including glioma. MHC-1 is the main immunogenic factor responsible for auto-recognition by the host immune system. As a result, it's believed that using patient-specific EVs for therapeutic purposes will result in minimal or no immunological response [89].

EVs are a two-pronged system: in some cases, they can be employed to treat tumour cells, but in others, they can aid in the progression of cancer. Based on this knowledge, numerous techniques have been developed with the goal of suppressing or removing EVs from the circulation of patients. EV release pathways have been found to be activated by elevated Ca^2 +^ ions in a number of cancer cell lines, which is then activated by specific protein receptors and binding proteins like Munc 13-4, a Ca^2 +^ dependent soluble Nethylmaleimide–sensitive factor attachment protein (SNAP) receptor and Rab binding protein. As a result, reduction of mammalian uncoordinated 13-4 **(**Munc 13-4) would allow oncogenic EV secretion to be suppressed [90]. Lastly, technologies have also been developed that allow EVs to be filtered from the patient’s complete circulatory system. Using EV-binding lectins and antibodies, rapid extracorporeal capture and preservation of selected vesicles from the systemic circulation is possible [91].

## Conclusions

The discovery of EVs’ distinctive structure, various cargo components, potential roles in cancer progression, and modulation of many molecular signaling pathways is presently a hot study topic in the field of cancer theragnostics. EVs are a one-of-a-kind, highly efficient inter- and intracellular communication system that is involved in the transmission of a variety of physiologically active compounds that are known to play critical roles in not just health but also the onset of numerous diseases, including cancer. EVs are involved in an elaborated mechanism of communication, membrane trafficking, and movement of nucleic acids, oncogenic, and immunological modulatory substances between the many cellular components of the tumour cell and the surrounding stroma in a heterogeneous GBM environment. Despite the promising roles of EVs as potential diagnostic and therapeutic modality for GBM, currently few studies have been devoted to evaluating their potential theragnostic roles for GBM at the clinical level. Even though the involvement of EVs in the progression of GBM has been extensively recognized, there are still significant impediments to EV utilization for diagnostic and therapeutic purposes in GBM. Isolation, subtyping, enrichment, cargo loading, and imparting target specificity to EVs are all examples. Overcoming these barriers will pave the way for EV-based medicines to become a routine modality of treatment for glioma patients in the future.

## Data Availability

All data are available in the manuscript.
